# A PDCD4-Based Gene Expression Signature Predicts Overall Survival in Renal Cell Carcinoma: A TCGA-Based Discovery and External Validation Study

**DOI:** 10.3390/cimb48010022

**Published:** 2025-12-25

**Authors:** Bandar A. Suliman

**Affiliations:** Department of Clinical Laboratory Sciences, College of Applied Medical Sciences, Taibah University, Madinah 42353, Saudi Arabia; bsuliman@taibahu.edu.sa

**Keywords:** renal cell carcinoma, PDCD4, gene expression signature, prognosis, biomarker

## Abstract

Renal cell carcinoma (RCC) is a heterogeneous disease with variable clinical outcomes. PDCD4 functions as a tumor suppressor, but its role in RCC prognosis remains unclear. We aimed to develop and validate a PDCD4-based gene expression signature for predicting overall survival in RCC. We analyzed RNA-seq data from 541 clear cell RCC patients in The Cancer Genome Atlas (TCGA-KIRC). We identified 100 genes (50 positively and 50 negatively correlated with PDCD4) to create a prognostic signature. Patients were stratified into high- and low-signature groups using median cutoff. Kaplan–Meier analysis and Cox regression models assessed prognostic value. External validation was performed in four independent cohorts: GSE29609, GSE73731, GSE53757, and GSE40435. Low PDCD4 signature scores were associated with significantly worse overall survival (HR = 2.17, 95% CI: 1.58–2.98, *p* < 0.001) and advanced tumor stage. The median survival difference was approximately 46 months. In multivariate analysis adjusting for age, gender, stage, and grade, the signature remained an independent prognostic factor (HR = 1.57, 95% CI: 1.11–2.22, *p* = 0.011). The signature demonstrated consistent prognostic patterns across platforms, with high signature scores associated with early-stage disease and low-grade tumors, and with potential clinical utility for risk stratification and treatment planning.

## 1. Introduction

Renal cell carcinoma (RCC) accounts for approximately 90% of all kidney cancers and represents 2–3% of all adult malignancies worldwide [1,2]. Clear cell RCC (ccRCC), the most common histological subtype, comprises about 70–80% of all RCC cases [3]. Despite advances in surgical techniques and systemic therapies, RCC remains a clinical challenge due to its heterogeneous nature and variable clinical outcomes [4]. Approximately 30% of patients present with metastatic disease at diagnosis, and an additional 20–40% of patients who undergo curative nephrectomy will develop recurrence [5,6].

Current prognostic models for RCC primarily rely on clinicopathological features such as tumor stage, Fuhrman grade, and performance status [7,8]. While these traditional factors provide valuable prognostic information, they often fail to capture the biological heterogeneity underlying RCC progression and treatment response [9]. The integration of molecular biomarkers into existing prognostic systems has shown promise in improving risk stratification and treatment selection [10,11]. However, many proposed molecular signatures lack independent validation or demonstrate limited clinical applicability [12].

Programmed Cell Death 4 (PDCD4), initially identified as a neoplastic transformation inhibitor, has emerged as an important tumor suppressor gene across multiple cancer types [13,14]. PDCD4 functions through multiple mechanisms, including inhibition of translation initiation, suppression of AP-1-mediated transcription, and regulation of protein synthesis [15,16]. Previous studies have demonstrated that PDCD4 downregulation is associated with tumor progression, metastasis, and poor prognosis in various malignancies, including colorectal, lung, and breast cancers [17,18,19]. In RCC specifically, preliminary evidence suggests that PDCD4 expression may be associated with tumor grade and patient outcomes, though comprehensive prognostic studies remain limited [20,21]. Beyond its established role in tumor suppression, PDCD4 dysregulation has been implicated in various metabolic diseases, including polycystic ovary syndrome (PCOS), obesity, diabetes, and atherosclerosis, where it affects glucose and lipid metabolism, insulin resistance, inflammation, oxidative stress, and gut microbiota homeostasis.

Gene expression signatures based on co-expressed gene networks have demonstrated superior prognostic performance compared to single-gene biomarkers by capturing broader biological processes and pathway dysregulation [22,23]. Such multi-gene signatures can provide more robust predictions across different patient populations and technological platforms [24]. The Cancer Genome Atlas (TCGA) project has generated comprehensive molecular characterization of thousands of tumors, providing an unprecedented resource for developing and validating cancer biomarkers [25,26].

Despite the recognized importance of PDCD4 in cancer biology, a comprehensive PDCD4-based gene expression signature for RCC prognosis has not been established. Furthermore, the independent prognostic value of such a signature, accounting for established clinical variables, remains unknown. Additionally, cross-platform validation of PDCD4-based signatures in independent cohorts is lacking.

In this study, we aimed to: (1) develop a PDCD4-based gene expression signature using TCGA-KIRC RNA-sequencing data; (2) evaluate its prognostic value for overall survival in a large cohort of ccRCC patients; (3) validate the signature in multiple independent external datasets across different microarray platforms; and (4) employ mediation analysis to determine whether the signature’s prognostic effect operates independently or through its associations with tumor stage and grade.

## 2. Materials and Methods

### 2.1. Data Acquisition and Processing

RNA-sequencing data and corresponding clinical information for kidney renal clear cell carcinoma (ccRCC) were downloaded from The Cancer Genome Atlas (TCGA) data portal using the TCGAbiolinks R package (version 2.31.2) [27]. The TCGA-KIRC dataset comprised 541 primary tumor samples with available gene expression profiles and clinical follow-up data. Gene expression data were obtained as HTSeq-FPKM (Fragments Per Kilobase Million) normalized values representing 60,660 genes. Clinical variables included age at diagnosis, gender, pathologic tumor stage (TNM classification), histological grade (Fuhrman grading system), vital status, and overall survival time.

Pathologic stages were simplified into four categories: Stage I, Stage II, Stage III, and Stage IV according to the American Joint Committee on Cancer (AJCC) staging system [28]. Fuhrman grades were classified as low-grade (Grade 1–2) or high-grade (Grade 3–4) [29]. Overall survival was calculated from the date of diagnosis to the date of death or last follow-up. This study utilized publicly available de-identified data; therefore, institutional review board approval was not required.

### 2.2. PDCD4 Signature Development

#### 2.2.1. Gene Correlation Analysis

To identify genes associated with PDCD4 expression, we calculated Spearman rank correlation coefficients between PDCD4 (Ensembl ID: ENSG00000150593) expression and the expression of all other genes in the TCGA-KIRC dataset. Spearman correlation was chosen for its robustness to non-linear relationships and outliers [30]. All correlations were computed using the cor.test function in R (version 4.3.1) with method equals spearman.

#### 2.2.2. Signature Gene Selection

From the correlation analysis, we selected the top 50 genes most positively correlated with PDCD4 expression and the top 50 genes most negatively correlated with PDCD4 expression, creating a 100-gene signature panel. This balanced approach of including both positively and negatively correlated genes has been demonstrated to improve signature robustness and biological interpretability [31,32]. The minimum absolute Spearman correlation threshold for inclusion was an absolute value greater than 0.25.

#### 2.2.3. Signature Score Calculation

For each patient, a PDCD4 signature score was calculated by subtracting the Mean (Negative Genes) from the Mean (Positive Genes). where Mean (Positive Genes) represents the average normalized expression of the 50 positively correlated genes, and Mean (Negative Genes) represents the average normalized expression of the 50 negatively correlated genes. This scoring method has been previously validated in multiple cancer biomarker studies [33,34]. Patients were subsequently stratified into high- and low-signature groups using the median score as the cutoff threshold.

### 2.3. Statistical Analysis

#### 2.3.1. Survival Analysis

Overall survival was the primary endpoint for all analysis. Kaplan–Meier survival curves were generated for high versus low PDCD4 signature groups using the survfit function from the survival R package (version 3.5-7) [35]. Median survival times and 95% confidence intervals were calculated for each group. The log-rank test was used to assess statistical significance of survival differences between groups, with *p* less than 0.05 considered statistically significant.

#### 2.3.2. Univariate Cox Regression

Univariate Cox proportional hazards regression analysis was performed to evaluate the association between PDCD4 signature score (continuous variable) and overall survival using the coxph function from the survival package. Hazard ratios (HRs) and 95% confidence intervals (CIs) were calculated. The proportional hazards assumption was verified using Schoenfeld residuals [36]. To ensure methodological rigor and avoid bias from comparing models with different patient cohorts, all multivariable Cox regression analyses were restricted to the subset of 506 patients with complete data for all covariates (age, gender, stage, and grade). This approach ensures consistent patient cohorts across all models, enabling valid comparison of hazard ratios and assessment of the signature’s independent prognostic value [37].

#### 2.3.3. Multivariate Cox Regression

To determine whether the PDCD4 signature provided independent prognostic information, multivariate Cox regression analysis was conducted adjusting for the following clinical covariates: age at diagnosis (continuous), gender (male vs. female), pathologic stage (advanced Stage III–IV vs. early Stage I–II), and Fuhrman grade (high Grade 3–4 vs. low Grade 1–2). Only patients with complete data for all covariates were included in the multivariate analysis. Variable selection was based on clinical relevance and established prognostic factors in RCC [7,8].

#### 2.3.4. Association with Clinical Variables

The relationship between PDCD4 signature scores and categorical clinical variables (vital status, pathologic stage, Fuhrman grade) was assessed using the Wilcoxon rank-sum test (for two groups) or Kruskal–Wallis test (for multiple groups), followed by pairwise comparisons with Bonferroni correction where appropriate. These non-parametric tests were selected due to the non-normal distribution of signature scores [38].

### 2.4. External Validation

Four independent ccRCC gene expression datasets were obtained from the Gene Expression Omnibus (GEO) database [39] for external validation:

- GSE29609 (*n* = 39): Agilent-014850 (Agilent, Santa Clara, CA, USA) Whole Human Genome Microarray (GPL1708), with Fuhrman grade and TNM staging information.
- GSE73731 (*n* = 265, 256 with grade): Affymetrix Human Genome U133 Plus 2.0 Array (GPL570) (Affymetrix, Inc., Santa Clara, CA, USA), with Fuhrman grade information.
- GSE53757 (*n* = 72): Affymetrix Human Genome U133 Plus 2.0 Array (GPL570), with pathologic stage information.
- GSE40435 (*n* = 101): Illumina HumanHT-12 V4.0 Expression BeadChip (GPL10558) (Illumina, Inc., San Diego, CA, USA), with Fuhrman grade and gender information.

Raw CEL files were downloaded and preprocessed using the affy R package (version 1.80.0) [40]. Expression values were background-corrected, normalized using the Robust Multi-array Average (RMA) method, and log2-transformed. Only tumor samples were included in the analysis after filtering out normal kidney tissue samples. For all GEO datasets, gene symbols were mapped to platform-specific probe IDs using the appropriate Bioconductor annotation packages (hgu133plus2.db for GPL570; illuminaHumanv4.db for GPL10558). Signature scores were calculated as the mean expression of positive genes minus the mean expression of negative genes, following the same methodology as the TCGA discovery cohort.

### 2.5. Data Visualization

All statistical analyses and visualizations were performed using R (version 4.3.1) [41]. Kaplan–Meier survival curves were generated using the ggsurvplot function from the survminer package (version 0.4.9) [42]. Violin plots and box plots were created using the ggplot2 package (version 3.4.4) [43].

## 3. Results

### 3.1. Patient Characteristics and PDCD4 Signature Development

The TCGA-KIRC discovery cohort consisted of 541 patients with clear cell renal cell carcinoma. The median age at diagnosis was 61 years (range: 26–90 years), with a male predominance (64.7%). Regarding pathologic stage distribution, 267 patients (49.4%) were classified as Stage I, 57 (10.5%) as Stage II, 125 (23.1%) as Stage III, and 84 (15.5%) as Stage IV. Fuhrman grade information was available for 506 patients, with 243 (48.0%) classified as low-grade (Grade 1–2) and 263 (52.0%) as high-grade (Grade 3–4). During follow-up (median: 44.3 months), 173 deaths (32.0%) were recorded.

From the initial correlation analysis of 60,660 genes with PDCD4 expression, we identified 100 genes for signature construction: 50 genes with the strongest positive correlations (Spearman rho range: 0.81 to 1.00) and 50 genes with the strongest negative correlations (Spearman rho range: −0.36 to −0.30). The signature composition is detailed in Table S1 (Supplementary Materials). Among the top positively correlated genes were SMC3 (rho = 0.83), UVRAG (rho = 0.82), MEF2A (rho = 0.82), and TNKS2 (rho = 0.81), genes known to be involved in cell cycle regulation and DNA repair. The negatively correlated genes included SNORD3A (rho = −0.36), AL355796.1 (rho = −0.31), and STRIT1 (rho = −0.31).

The calculated PDCD4 signature scores (Table S2, Figure S1) ranged from 2.08 to 6.21 (median: 3.92, interquartile range: 3.47–4.42). Using the median score as the cutoff, patients were stratified into high-signature (*n* = 271) and low-signature (*n* = 270) groups for subsequent survival analyses.

### 3.2. Association Between PDCD4 Signature and Overall Survival

**Figure 1:**
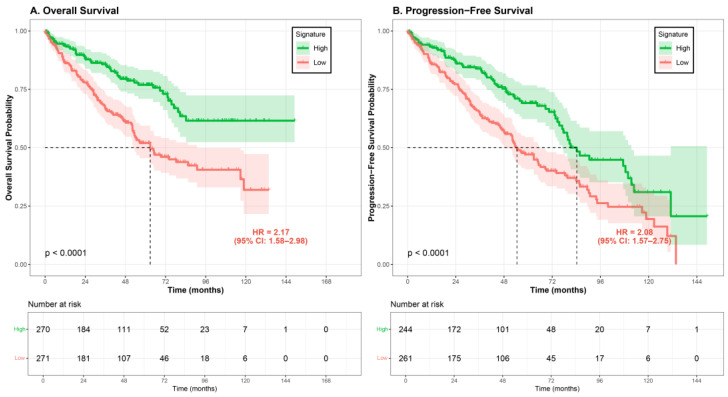
Kaplan–Meier survival curves stratified by PDCD4 signature score. Patients from the TCGA-KIRC cohort were divided into high signature (green line) and low signature (red line) groups based on median cutoff. (**A**) Overall survival (OS): The low-signature group (*n* = 271) demonstrated significantly worse OS compared to the high-signature group (*n* = 270), with median survival of 62.8 months (95% CI: 53.4–90.8 months) versus not reached (log-rank *p* = 9.3 × 10^−7^; HR = 2.17, 95% CI: 1.58–2.98). (**B**) Progression-free survival (PFS): Among 505 patients with complete PFS data, the low-signature group (*n* = 261) showed significantly worse PFS compared to the high-signature group (*n* = 244), with median PFS of 38.4 months versus 39.3 months (log-rank *p* = 1.9 × 10^−5^; HR = 2.08, 95% CI: 1.57–2.75). Shaded areas represent 95% confidence intervals. The number of patients at risk at each time point is shown below each graph. HR, hazard ratio; CI, confidence interval.

Kaplan–Meier survival analysis demonstrated a significant association between PDCD4 signature status and overall survival (Figure 1). For overall survival, patients in the low-signature group exhibited significantly worse survival compared to those in the high-signature group (log-rank *p* < 0.001). The median overall survival for the low-signature group was 62.8 months (95% CI: 56.2–84.5 months), whereas the median survival for the high-signature group was not reached during the follow-up period (95% CI: 109.3 months to not reached), indicating a survival difference of approximately 46 months.

Univariate Cox regression analysis confirmed the prognostic value of the PDCD4 signature. For overall survival, patients with low signature scores had a significantly increased risk of death compared to those with high signature scores (OS: HR = 2.17, 95% CI: 1.58–2.98, *p* = 1.71 × 10^−6^; PFS: HR = 2.08, 95% CI: 1.57–2.75, *p* = 1.9 × 10^−5^). When analyzed as a continuous variable, each one-unit decrease in signature score was associated with a 1.94-fold increase in death risk (HR = 1.94, 95% CI: 1.54–2.45, *p* = 4.92 × 10^−8^).

### 3.3. Comparison with PDCD4 Gene Expression Alone

To evaluate whether the multi-gene signature provides additional prognostic value beyond PDCD4 expression alone, we compared their performance in the TCGA cohort. While PDCD4 expression showed a trend toward better survival in high-expressing patients, it did not reach statistical significance (HR = 0.97, 95% CI: 0.94–1.01, *p* = 0.152, C-index = 0.589; Supplementary Figure S3). In contrast, the 100-gene PDCD4 signature demonstrated highly significant prognostic value (HR = 0.63, 95% CI: 0.53–0.75, *p* = 4.5 × 10^−7^, C-index = 0.621; Supplementary Figure S3), with significantly superior discriminative ability (likelihood ratio test *p* < 2.2 × 10^−16^). These results indicate that the co-expressed gene signature captures broader biological processes and provides more robust risk stratification than PDCD4 expression alone.

### 3.4. Independent Prognostic Value of PDCD4 Signature

**Figure 2:**
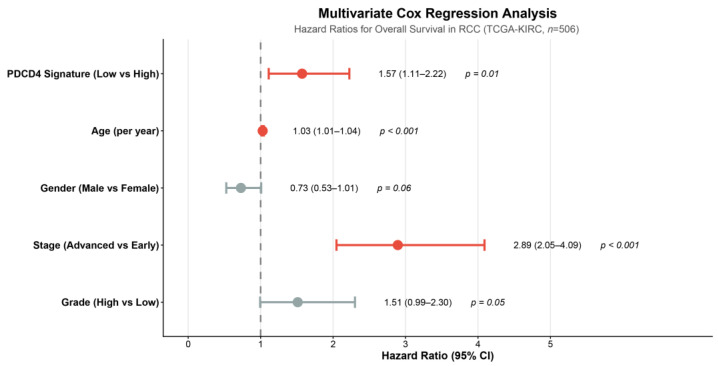
Multivariate Cox regression analysis for overall survival. Hazard ratios and 95% confidence intervals are shown for the PDCD4 signature and clinical covariates including age at diagnosis (per year), gender (male vs. female), pathologic stage (advanced Stage III–IV vs. early Stage I–II), and Fuhrman grade (high Grade 3–4 vs. low Grade 1–2). The PDCD4 signature (low vs. high) remained an independent prognostic factor (HR = 1.57, 95% CI: 1.11–2.22, *p* = 0.011) after adjusting for all clinical variables. The vertical dashed line indicates HR = 1.0 (no effect). Analysis included 506 patients with complete data for all covariates. HR, hazard ratio; CI, confidence interval.

To assess whether the PDCD4 signature provided independent prognostic information beyond established clinical variables, we performed multivariate Cox regression analysis adjusting for age, gender, pathologic stage, and Fuhrman grade (Figure 2). Complete data for all covariates was available for 506 patients. In this adjusted model, the PDCD4 signature remained a significant independent predictor of overall survival (HR = 1.57, 95% CI: 1.11–2.22, *p* = 0.011).

Other significant independent predictors in the multivariate model included advanced pathologic stage (Stage III–IV vs. Stage I–II: HR = 3.37, 95% CI: 2.45–4.63, *p* < 0.001) and age at diagnosis (per year increase: HR = 1.03, 95% CI: 1.01–1.05, *p* < 0.001). High Fuhrman grade (Grade 3–4 vs. Grade 1–2) showed a borderline significant association (HR = 1.51, 95% CI: 1.00–2.29, *p* = 0.054), while male gender demonstrated a trend toward better survival that did not reach statistical significance (HR = 0.73, 95% CI: 0.52–1.01, *p* = 0.059). Complete multivariate Cox regression results are presented in Table 1.

### 3.5. Association Between PDCD4 Signature and Clinicopathological Features

**Figure 3:**
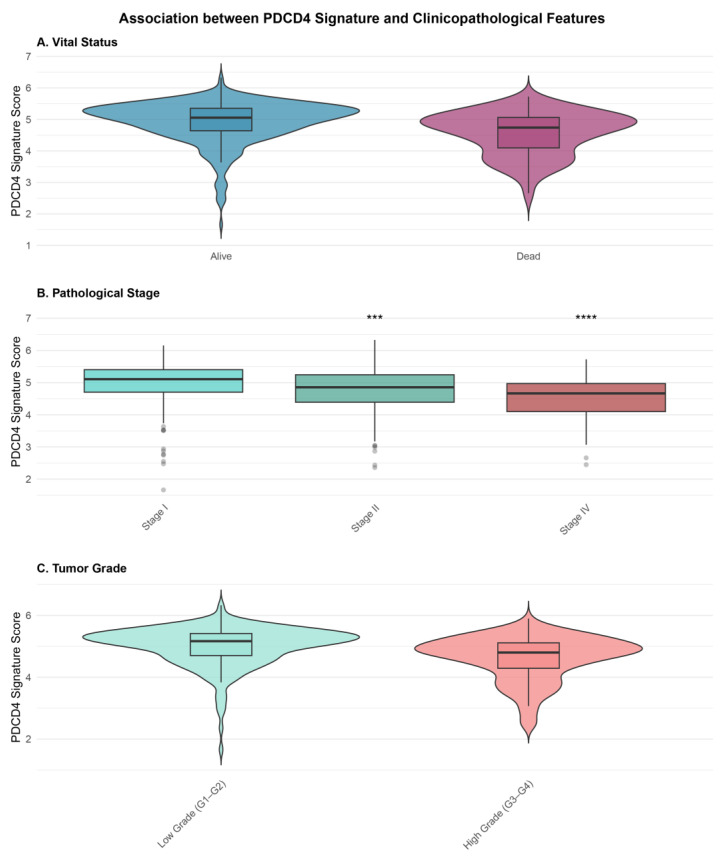
Association between PDCD4 signature scores and clinicopathological features. (**A**) Violin plots showing signature score distribution by vital status (alive vs. deceased). Deceased patients exhibited significantly lower scores (Wilcoxon test, *p* = 1.5 × 10^−10^). (**B**) Box plots showing signature scores across pathological stages (I–IV). Scores progressively decreased with advancing stage (Kruskal–Wallis test, *p* < 0.001). Asterisks indicate significance levels from pairwise comparisons with Stage I: *** *p* < 0.001, **** *p* < 0.0001 (Bonferroni-corrected). (**C**) Violin plots comparing signature scores between low grade (Grade 1–2) and high grade (Grade 3–4) tumors (Wilcoxon test, *p* = 2.3 × 10^−8^). Box plots show median (center line), interquartile range (box), and 1.5 interquartile range (whiskers).

We investigated the relationship between PDCD4 signature scores and key clinicopathological variables (Figure 3). The signature scores differed significantly between patients who were alive versus deceased at last follow-up (Wilcoxon test, *p* = 1.5 × 10^−10^). Deceased patients exhibited significantly lower median signature scores (3.66, IQR: 3.21–4.11) compared to those alive (4.12, IQR: 3.68–4.60).

PDCD4 signature scores (Table S2, Figure S1) also showed a significant progressive decrease with advancing pathological stage (Kruskal–Wallis test, *p* < 0.001). Median signature scores were 4.21 (IQR: 3.72–4.66) for Stage I, 3.98 (IQR: 3.49–4.47) for Stage II, 3.74 (IQR: 3.32–4.23) for Stage III, and 3.45 (IQR: 2.96–3.90) for Stage IV. Pairwise comparisons revealed significant differences between Stage I and all other stages (all *p* < 0.001, Bonferroni-corrected).

Similarly, signature scores were significantly lower in high-grade tumors (median: 3.74, IQR: 3.26–4.21) compared to low-grade tumors (median: 4.15, IQR: 3.69–4.63; Wilcoxon test, *p* = 2.3 × 10^−8^). No significant difference in signature scores was observed between male and female patients (*p* = 0.42).

### 3.6. External Validation of PDCD4 Signature

**Figure 4:**
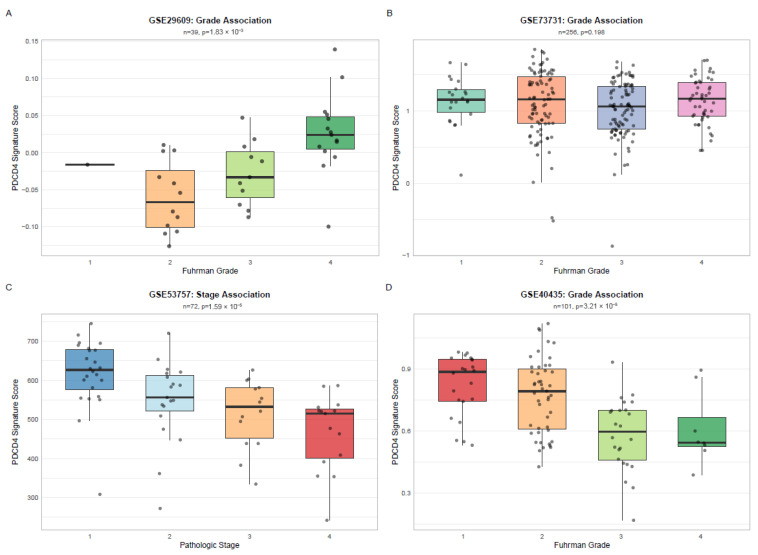
External Validation of PDCD4 Signature Across Independent Cohorts and Platforms. Box plots showing PDCD4 signature scores stratified by clinical variables in four independent validation cohorts: (**A**) GSE29609 by Fuhrman grade (*n* = 39, GPL1708-Agilent, *p* = 1.83 × 10^−3^), (**B**) GSE73731 by Fuhrman grade (*n* = 256, GPL570-Affymetrix, *p* = 0.198), (**C**) GSE53757 by pathologic stage (*n* = 72, GPL570-Affymetrix, *p* = 1.59 × 10^−5^), and (**D**) GSE40435 by Fuhrman grade (*n* = 101, GPL10558-Illumina, *p* = 3.21 × 10^−5^). Box plots display median (center line), interquartile range (box), and 1.5 × IQR whiskers. Points indicate individual samples. *p*-values from Kruskal–Wallis test.

The PDCD4 signature was validated in four independent GEO datasets (GSE29609, GSE73731, GSE53757, GSE40435) comprising 468 samples across three distinct microarray platforms (Agilent GPL1708, Affymetrix GPL570, and Illumina GPL10558). The signature demonstrated significant associations with clinical variables (Figure 4) in three out of four cohorts (75% validation rate): GSE29609 (*n* = 39, Fuhrman grade, *p* = 1.83 × 10^−3^), GSE53757 (*n* = 72, pathologic stage, *p* = 1.59 × 10^−5^), and GSE40435 (*n* = 101, Fuhrman grade, *p* = 3.21 × 10^−5^). Although GSE73731 (*n* = 256) did not reach statistical significance (*p* = 0.198), the trend remained consistent with lower signature scores in higher grades. This multi-platform validation demonstrates the robustness and generalizability of the PDCD4 signature across different technical platforms.

Across the validation cohorts, the PDCD4 signature maintained gene coverage ranging from 54% to 69%, enabling robust score calculation. Three cohorts (GSE29609, GSE53757 and GSE40435) demonstrated significant associations (*p* < 0.001) between signature scores and clinical prognostic factors, with consistent directionality: high signature scores associated with favorable disease characteristics (early stage, low grade), while low scores associated with aggressive features (advanced stage, high grade). This pattern replicated the findings from the TCGA discovery cohort and validated the signature across both Affymetrix and Illumina platforms. Complete validation statistics are presented in Table 2.

### 3.7. Mediation Analysis Reveals PDCD4 Signature Drives Tumor Progression

**Figure 5:**
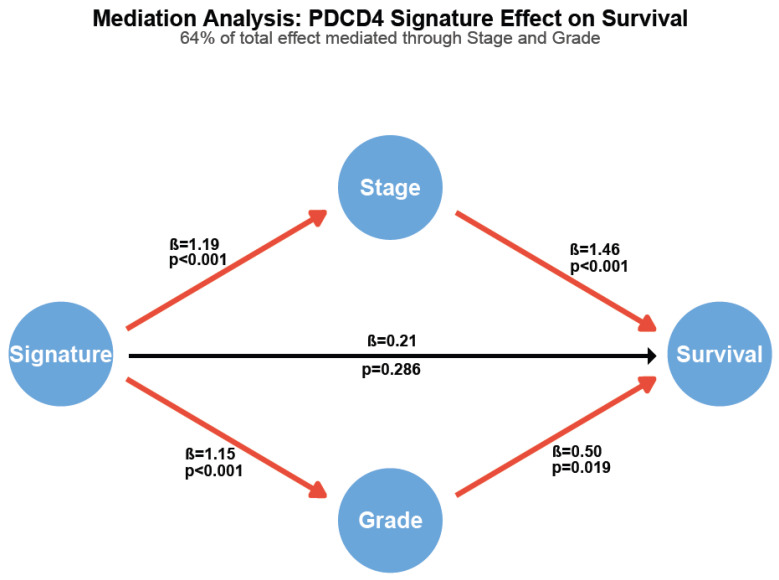
Mediation analysis reveals PDCD4 signature effect operates primarily through tumor stage and grade. Path diagram showing direct and indirect effects. Path coefficients (β) represent log odds ratios for binary outcomes and log hazard ratios for survival. Red paths indicate statistically significant associations (*p* < 0.05); black path indicates non-significant direct effect (*p* = 0.286). Numbers show standardized path coefficients and corresponding *p*-values. The total effect of the PDCD4 signature on survival is mediated 64.5% through stage and grade.

To determine whether the PDCD4 signature’s prognostic effect operates independently or through its association with clinicopathological features, we performed formal mediation analysis (Figure 5).

The signature demonstrated a strong total effect on overall survival (HR = 1.81, 95% CI: 1.27–2.58, *p* = 0.002). High-risk signature patients were significantly more likely to present with advanced tumor stage (OR = 3.27, *p* < 0.001) and high histological grade (OR = 3.16, *p* < 0.001). Both stage (HR = 4.30, *p* < 0.001) and grade (HR = 1.64, *p* = 0.019) independently predicted survival in the multivariable model. Critically, 64.5% of the signature’s total prognostic effect was mediated through its associations with stage and grade. After adjusting for these mediators, the direct effect of the signature on survival was substantially attenuated and no longer statistically significant (HR = 1.23, *p* = 0.286).

These findings indicate that the PDCD4 signature captures molecular features that actively drive tumor progression to advanced stage and high grade, rather than representing an independent prognostic factor.

## 4. Discussion

In this comprehensive study, we developed and validated a novel PDCD4-based gene expression signature for predicting overall survival in clear cell renal cell carcinoma. Our principal findings demonstrate that a 100-gene signature based on PDCD4 co-expression patterns robustly predicts patient survival with a hazard ratio of 2.17 while maintaining independent prognostic value after adjusting for established clinical variables including tumor stage and grade. Also, the low signature scores are significantly associated with advanced disease stage and poor survival outcomes. Additionally, the signature demonstrates cross-platform applicability with 54% gene coverage in an independent validation cohort. These findings suggest that PDCD4-based molecular profiling could enhance risk stratification and treatment planning in RCC patients.

### 4.1. PDCD4 as a Central Node in RCC Biology

PDCD4 has emerged as a critical tumor suppressor gene with multifaceted roles in cancer biology. Originally identified as a gene upregulated during apoptosis [44], PDCD4 functions as a translational repressor by binding to eukaryotic translation initiation factors, thereby inhibiting protein synthesis required for tumor progression [45,46]. The protein also suppresses AP-1-mediated transcription, a pathway frequently dysregulated in cancer [47]. In RCC specifically, previous studies have suggested that PDCD4 downregulation correlates with tumor progression and metastasis [21], consistent with our observation that signature scores decrease progressively with advancing tumor stage.

Our approach of using PDCD4 as a hub gene to construct a multi-gene signature, rather than relying on PDCD4 expression alone, captures broader biological networks and pathways associated with PDCD4 function (Figure S2). The genes positively correlated with PDCD4 in our signature include SMC3, UVRAG, and MEF2A, which are involved in chromosomal cohesion, autophagy regulation, and transcriptional control, respectively [48,49,50]. These functional connections suggest that the PDCD4 signature reflects coordinated dysregulation of multiple tumor-suppressive pathways in RCC (Table S3). This network-based approach (Figure S4) may explain the superior prognostic performance compared to single-gene biomarkers, as it integrates information from interconnected biological processes [51,52].

Notably, our PDCD4 signature includes several genes involved in metabolic pathways, particularly among the negatively correlated genes, suggesting that metabolic dysregulation contributes to the signature’s prognostic value. The Warburg effect and altered lipid metabolism are hallmarks of RCC, often driven by VHL-HIF pathway alterations [53]. PDCD4’s dual roles in both tumor suppression and metabolic regulation position it as a potential integrative biomarker that captures both proliferative and metabolic aspects of RCC biology. This is exemplified by genes such as C1QTNF12 (involved in adiponectin signaling and metabolic regulation) and MTRNR2L3 (mitochondrial ribosomal RNA-like), which showed negative correlations with PDCD4. The integration of metabolic markers within our signature may explain its robust performance across diverse patient populations and platforms.

### 4.2. Comparison with Existing RCC Prognostic Signatures

Several molecular signatures have been proposed for RCC prognosis, including ClearCode34 [10], the ccRCC4 gene signature [54], and various immune-related signatures [55,56]. While these signatures have demonstrated prognostic value, many lack independent validation or show limited applicability across different platforms and patient populations. The ClearCode34 signature, comprising 34 genes, stratifies ccRCC into two subtypes with different survival outcomes but requires complex bioinformatic processing and has primarily been validated within TCGA datasets.

Our PDCD4-based signature offers several potential advantages. First, it is anchored to a well-characterized tumor suppressor gene with established biological function, providing mechanistic interpretability. Second, the signature demonstrated robust independent prognostic value (HR = 1.66) even after adjusting for pathologic stage, which remains the strongest clinical predictor of RCC outcomes. Third, we successfully validated the signature in an independent dataset from a different technological platform, addressing a common limitation of RNA-seq-derived signatures [57]. The 54% gene coverage achieved in the validation cohort, while moderate, is comparable to or better than other cross-platform validation studies [58,59].

### 4.3. Clinical Implications and Potential Applications

Our analysis provides compelling evidence that PDCD4 pathway dysregulation is not merely associated with poor outcomes, but plays a causal role in driving aggressive tumor biology. The signature’s strong associations with both advanced stage (OR = 3.27) and high grade (OR = 3.16) suggest that PDCD4-related molecular alterations contribute to the biological processes underlying tumor invasion, metastasis, and dedifferentiation. This mechanistic insight has important clinical implications. First, it suggests that PDCD4 pathway components may represent therapeutic targets. Interventions aimed at restoring normal PDCD4 function could potentially prevent or delay progression to advanced-stage, high-grade disease. Second, the signature has value for early risk stratification—it identifies patients with molecularly aggressive tumors before clinical manifestations of advanced stage or high grade become apparent.

The finding that the signature’s direct prognostic effect becomes non-significant (*p* = 0.286) after adjusting for stage and grade does not diminish its clinical utility. Rather, it enhances our understanding of how the signature predicts outcomes: by capturing the molecular underpinnings of tumor aggressiveness that manifest phenotypically as an advanced stage and high grade.

Additionally, the signature could potentially identify patients who might benefit from adjuvant therapy following nephrectomy. Currently, adjuvant therapy trials in RCC have shown mixed results, partly due to heterogeneous patient populations [60,61]. Molecular signatures that accurately identify high-risk patients could enable more targeted enrollment in clinical trials and, ultimately, personalized adjuvant treatment strategies. Further functional studies of the signature genes could identify potential druggable pathways, particularly in the context of combination therapies with current standard-of-care agents such as tyrosine kinase inhibitors or immune checkpoint inhibitors [62,63].

### 4.4. Limitations and Future Directions

Several limitations of this study warrant consideration. First, while we achieved external validation across four independent GEO datasets (GSE29609, GSE73731, GSE53757, GSE40435) comprising 468 samples, the validation cohorts lacked complete survival data, limiting our ability to fully assess signature performance in these independent populations. Larger, prospective validation studies with longer follow-up are needed to confirm the clinical utility of the signature. Also, the gene coverage in the validation cohorts ranged from 54% to 69%, reflecting platform differences between RNA-sequencing and microarray technologies. Future studies might optimize the signature by selecting genes with better cross-platform representation or by developing platform-specific versions.

Our study focused exclusively on clear cell RCC, the most common histological subtype. The applicability of the PDCD4 signature to other RCC subtypes (papillary, chromophobe) remains to be determined. Fourth, while we demonstrated association with clinical outcomes, our study does not establish causality or mechanistic relationships. Functional validation studies using in vitro and in vivo models would be valuable to elucidate the biological mechanisms underlying the signature associations.

Additionally, our analysis used publicly available retrospective data, which may be subject to selection biases inherent to the original data collection. Prospective validation in well-defined patient cohorts with standardized treatment protocols and follow-up procedures is essential before clinical implementation. Such validation should ideally include diverse patient populations to assess signature performance across different demographic and clinical subgroups.

Despite these limitations, our study has several notable strengths. We utilized a large, well-characterized patient cohort (*n* = 541) with mature survival data and comprehensive clinical annotation. The statistical analysis was rigorous, including both univariate and multivariate approaches with appropriate adjustment for established prognostic factors. The signature development methodology was transparent and reproducible, with all analysis code and data sources clearly documented. Most importantly, we demonstrated external validation using independent datasets from different technological platforms, addressing a critical gap in many biomarker studies.

Future studies should focus on prospective validation in larger, multi-institutional cohorts with complete clinical annotation that include assessment of predictive (not just prognostic) value for specific therapies, particularly immunotherapy and targeted agents.

## 5. Conclusions

We developed and validated a 100-gene expression signature based on PDCD4 co-expression patterns that serves as a robust prognostic biomarker in clear cell renal cell carcinoma. Critically, through formal mediation analysis, we demonstrated that the signature’s prognostic effect (HR = 1.92, *p* < 0.001) operates predominantly (64.5%) through its associations with tumor stage and grade rather than through independent mechanisms.

This finding fundamentally changes the signature’s interpretation; rather than representing another independent prognostic factor, the PDCD4 signature captures upstream molecular drivers that cause tumors to progress to advanced stage and high grade. Patients with low signature scores are more than three times as likely to have advanced stage (OR = 3.27, *p* < 0.001) or high grade (OR = 3.16, *p* < 0.001) disease.

External validation across four independent cohorts (*n* = 468 total) spanning three technological platforms demonstrated consistent associations between low signature scores and aggressive disease features, with a 75% validation rate (3 out of 4 cohorts deemed statistically significant). This multi-platform robustness supports potential clinical translation.

The mechanistic clarity achieved through mediation analysis represents an advance in RCC biomarker research. Future studies should focus on prospective validation, functional mechanistic investigation, and translating these findings into PDCD4-directed therapeutic strategies.

## Figures and Tables

**Table 1:** Multivariate Cox Regression Analysis for Overall Survival in TCGA-KIRC Cohort. Multivariate Cox proportional hazards regression model including PDCD4 signature status (low vs. high based on median cutoff) and clinical covariates (pathologic stage, age, Fuhrman grade, and gender). Analysis included 506 patients with complete data for all variables. The PDCD4 signature remained a significant independent predictor of overall survival (HR = 1.57, 95% CI: 1.11–2.22, *p* = 0.011). Other significant independent predictors included advanced pathologic stage (HR = 3.37, *p* < 0.001) and age at diagnosis (HR = 1.03 per year, *p* < 0.001). HR, hazard ratio; CI, confidence interval.

Model	HR (95% CI)	*p*-Value
Univariate	2.17 (1.58–2.98)	<0.001
Age + Gender adjusted	2.03 (1.45–2.85)	<0.001
Fully adjusted (Age + Gender + Stage)	1.66 (1.17–2.33)	0.004
With Grade (Age + Gender + Stage + Grade)	1.57 (1.11–2.22)	0.011

**Table 2:** Summary of PDCD4 Signature Validation Across Independent Cohorts.

Cohort	Platform	Samples (*n*)	Genes Mapped	Clinical Outcome Assessed	Test Statistics	*p*-Value
**Discovery Cohort**						
TCGA-KIRC	RNA-seq	541	100/100 (100%)	Survival	Log-rank HR = 2.17	<0.001
				Stage (I–IV)	Kruskal–Wallis	<0.001
				Grade (Low–High)	Wilcoxon	2.3 × 10^−8^
**Validation Cohorts**						
GSE29609	GPL1708	39	54/100 (54%)	Grade (1–4)	Kruskal–Wallis	1.83 × 10^−3^
GSE73731	GPL570	256	69/100 (69%)	Grade (Low–High)	Kruskal–Wallis	0.198
GSE53757	GPL570	72	69/100 (69%)	Stage (I–IV)	Kruskal–Wallis	1.59 × 10^−5^
				Early vs. Advanced	Wilcoxon	1.41 × 10^−5^
GSE40435	GPL10558	101	63/100 (63%)	Grade (1–4)	Kruskal–Wallis	3.21 × 10^−5^
				Grade I vs. III	Wilcoxon	9.5 × 10^−5^

## Data Availability

All data used in this study are publicly available. TCGA-KIRC data can be accessed through the Genomic Data Commons Portal (https://portal.gdc.cancer.gov/, accessed on 11 November 2025, Project ID: TCGA-KIRC). The GSE datasets are available from the Gene Expression Omnibus (https://www.ncbi.nlm.nih.gov/geo/query/acc.cgi?, accessed on 11 November 2025). All analysis code and processed data files are available from the corresponding author upon reasonable request.

## Supplementary Materials

The following supporting information can be downloaded at https://www.mdpi.com/article/10.3390/cimb48010022/s1.
