# Supplementary material for: A PDCD4-Based Gene Expression Signature Predicts Overall Survival in Renal Cell Carcinoma: A TCGA-Based Discovery and External Validation Study
Source: Curr Issues Mol Biol. 2025 Dec 25;48(1):22. doi: 10.3390/cimb48010022 (PMC12840172; doi:10.3390/cimb48010022)
Supplement: Supplementary file 1 [file cimb-48-00022-s001.zip › caption.pdf]

current issues in molecular biology

Table S1: Complete List of 100 PDCD4 Signature Genes Comprehensive listing of all 100 genes in the PDCD4 prognostic signature, ranked by absolute Spearman correlation coefficient with PDCD4 expression. The table includes gene symbols, correlation coefficients, and direction of correlation (positive/negative). All correlations were calculated using TCGA-KIRC RNA-seq data (n = 533 samples). Genes with positive correlations indicate co-expression with PDCD4, while negative correlations indicate inverse expression patterns. These 100 genes were used to calculate the PDCD4 signature score for each patient;

Table S2: PDCD4 Signature Scores for GEO Validation Cohort Patient-level PDCD4 signature scores for the independent GEO validation cohort (n = 39 ccRCC patients). Signature scores were calculated as the weighted sum of expression values for the 100 signature genes, with weights derived from TCGA training cohort correlations. Patients were stratified into high-risk and low-risk groups based on median signature score;

Table S3: Functional characterization of top correlated genes in the PDCD4-based prognostic signature. The table presents the 10 most positively correlated (co-activated) and 10 most negatively correlated (inversely regulated) genes with PDCD4 from the 100-gene prognostic signature. For each gene, the following information is provided: gene symbol, gene type (protein-coding, non-coding RNA, or pseudogene), Ensembl gene ID, OMIM ID (where available; non-coding RNAs and pseudogenes typically lack OMIM entries), correlation direction with PDCD4, primary biological pathway involvement, functional description based on current literature;

Figure S1: Distribution of PDCD4 Signature Scores in TCGA-KIRC Histogram showing the distribution of PDCD4 signature scores across all TCGA-KIRC samples (n = 541). The signature score represents the weighted expression of 100 PDCD4-correlated genes. The red dashed line indicates the median signature score (4.96), which was used as the cutoff to stratify patients into high-risk and low-risk groups. The distribution demonstrates continuous variation in the PDCD4 signature across clear cell renal cell carcinoma samples, supporting the biological relevance of this gene expression pattern;

Figure S2: Top 40 PDCD4-Correlated Genes plot showing the Spearman correlation coefficients for the top 20 positively and top 20 negatively correlated genes with PDCD4 expression. Blue bars indicate positive correlations (co-expression with PDCD4), while purple bars indicate negative correlations (inverse expression patterns). Genes are ordered by correlation strength. These genes represent the strongest associations with PDCD4 within the 100-gene prognostic signature and include genes involved in chromosomal organization (SMC3), autophagy (UVRAG), transcriptional regulation (MEF2A), and other cellular processes relevant to cancer biology;

Figure S3: Comparison of prognostic performance between PDCD4 gene expression alone and the PDCD4-based gene signature. (A) Kaplan-Meier survival curves stratified by PDCD4 gene expression (median dichotomization). PDCD4 expression alone showed no significant association with overall survival (Log-rank p=0.152, HR=0.97, C-index=0.589). (B) Kaplan-Meier survival curves stratified by the 100-gene PDCD4 signature score. The signature demonstrated highly significant prognostic value (Log-rank $p=4.5\times10^{-7}$, HR=0.63, C-index=0.621). The signature significantly outperformed PDCD4 alone (likelihood ratio test $p<2.2\times10^{-16}$), demonstrating superior discriminative ability through integration of 100 co-expressed genes;

Figure S4: Network schematic showing PDCD4 (purple hub) and its co-regulated genes in RCC. Positively correlated genes (green nodes, green edges; r > 0.7) include tumor suppressors involved in chromosome cohesion (SMC3), autophagy (UVRAG), and transcriptional regulation (MEF2A), representing tumor-suppressive pathways. Negatively correlated genes (orange nodes, orange edges) include non-coding RNAs (AL355796.1), pseudogenes (RPS23P6), and metabolic regulators (C1QTNF12), representing oncogenic pathways. This coordinated gene expression network forms the mechanistic basis of the PDCD4 prognostic signature in renal cell carcinoma.
