# Supplementary material for: A PDCD4-Based Gene Expression Signature Predicts Overall Survival in Renal Cell Carcinoma: A TCGA-Based Discovery and External Validation Study
Source: Curr Issues Mol Biol. 2025 Dec 25;48(1):22. doi: 10.3390/cimb48010022 (PMC12840172; doi:10.3390/cimb48010022)
Supplement: Supplementary file 1 [file cimb-48-00022-s001.zip › Supp_Figure_S3.pdf]

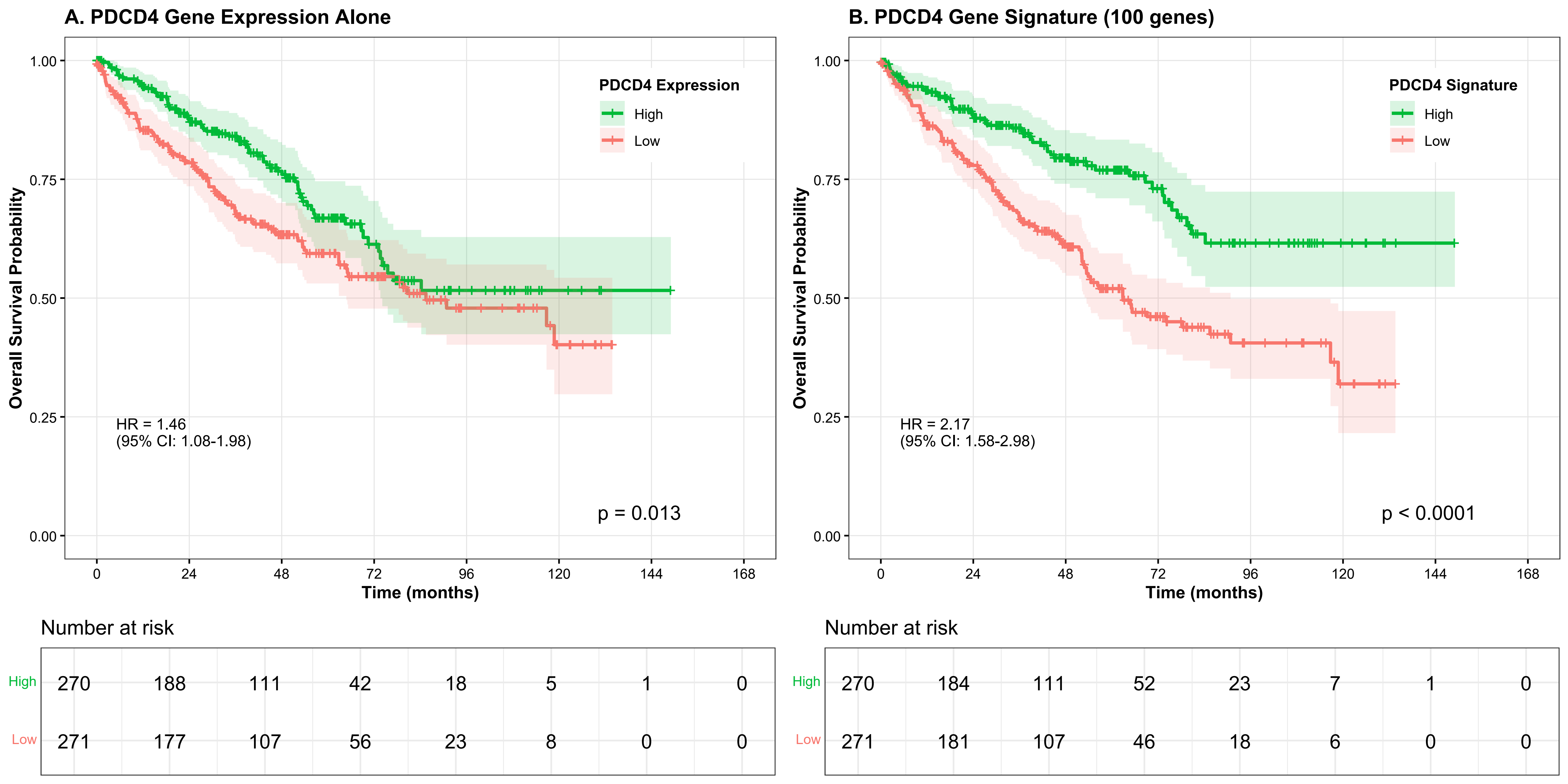

A. PDCD4 Gene Expression Alone
Overall Survival Probability
1.00
0.75
0.50
0.25
0.00
PDCD4 Expression
High
Low
HR = 1.46
(95% CI: 1.08-1.98)
p = 0.013
0
24
48
72
96
120
144
168
Time (months)
Number at risk
High 270 188 111 42 18 5 1 0
Low 271 177 107 56 23 8 0 0
B. PDCD4 Gene Signature (100 genes)
Overall Survival Probability
1.00
0.75
0.50
0.25
0.00
PDCD4 Signature
High
Low
HR = 2.17
(95% CI: 1.58-2.98)
p < 0.0001
0
24
48
72
96
120
144
168
Time (months)
Number at risk
High 270 184 111 52 23 7 1 0
Low 271 181 107 46 18 6 0 0
