# Supplementary material for: A PDCD4-Based Gene Expression Signature Predicts Overall Survival in Renal Cell Carcinoma: A TCGA-Based Discovery and External Validation Study
Source: Curr Issues Mol Biol. 2025 Dec 25;48(1):22. doi: 10.3390/cimb48010022 (PMC12840172; doi:10.3390/cimb48010022)
Supplement: Supplementary file 1 [file cimb-48-00022-s001.zip › Supp_Figure_S4.pdf]

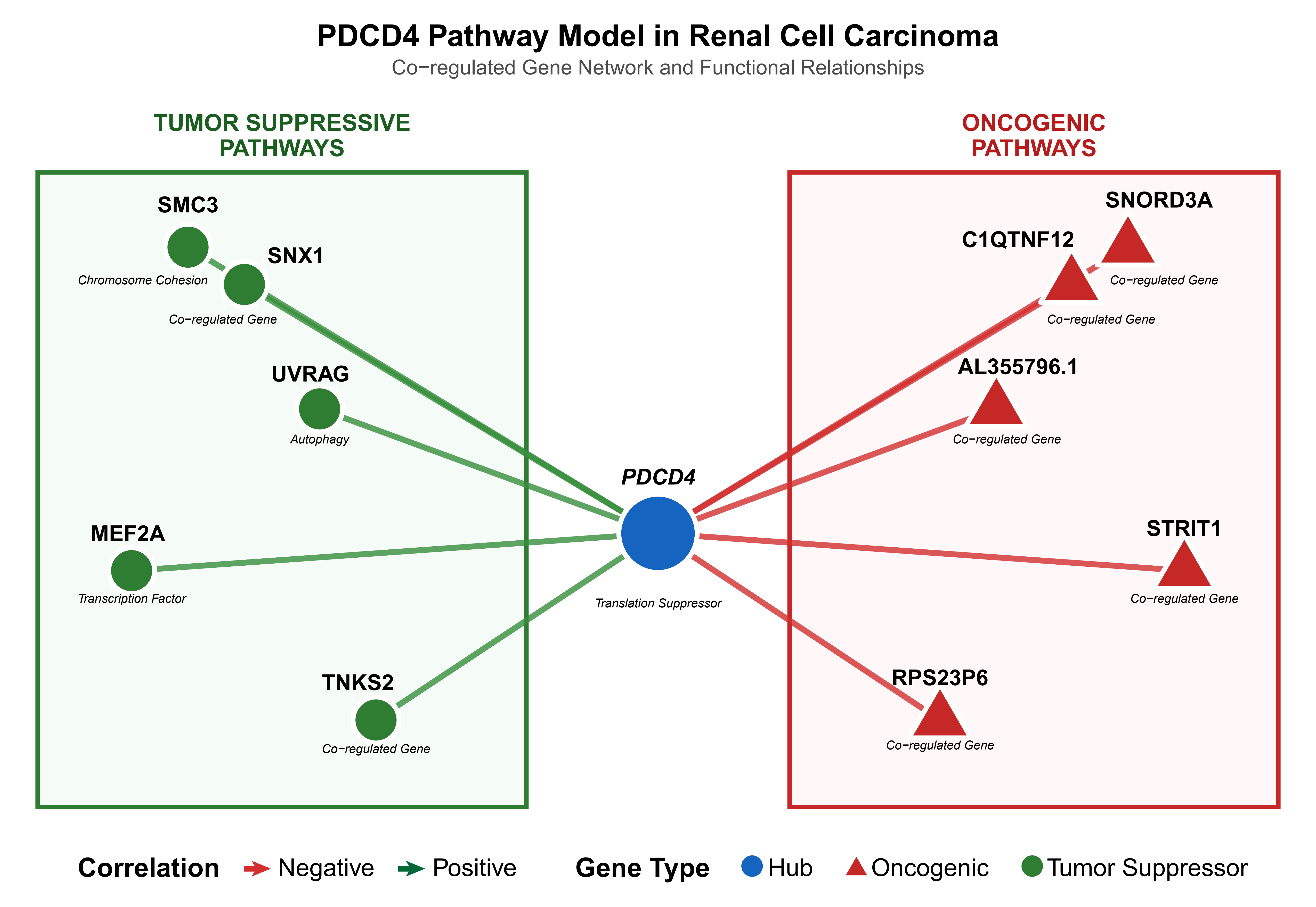
PDCD4 Pathway Model in Renal Cell Carcinoma
Co−regulated Gene Network and Functional Relationships
TUMOR SUPPRESSIVE PATHWAYS
ONCOGENIC PATHWAYS
SMC3
Chromosome Cohesion
SNX1
Co−regulated Gene
UVRAG
Autophagy
MEF2A
Transcription Factor
TNKS2
Co−regulated Gene
PDCD4
Translation Suppressor
SNORD3A
Co−regulated Gene
C1QTNF12
Co−regulated Gene
AL355796.1
Co−regulated Gene
STRIT1
Co−regulated Gene
RPS23P6
Co−regulated Gene
Correlation
Negative
Positive
Gene Type
Hub
Oncogenic
Tumor Suppressor
