# Supplementary figures and images for: A PDCD4-Based Gene Expression Signature Predicts Overall Survival in Renal Cell Carcinoma: A TCGA-Based Discovery and External Validation Study
Source: Curr Issues Mol Biol. 2025 Dec 25;48(1):22. doi: 10.3390/cimb48010022 (PMC12840172; doi:10.3390/cimb48010022)

# Distribution of PDCD4 Signature Scores in TCGA-KIRC

n = 541 samples | Median (red line) = 4.96

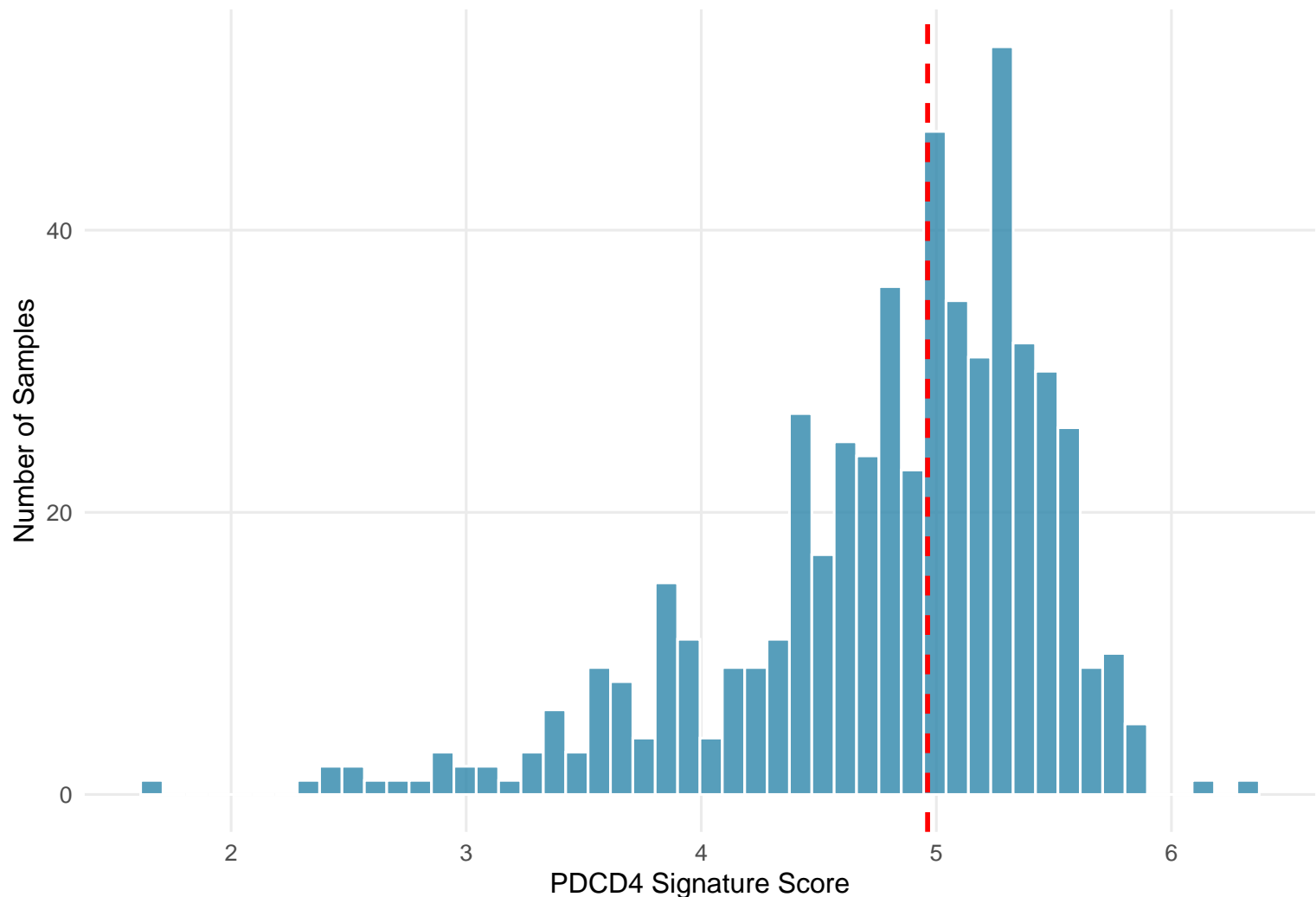

Supplement: Supplementary file 1 [file cimb-48-00022-s001.zip › Supp_Figure_S1.pdf]

Top 40 PDCD4–Correlated Genes

Top 20 positive and top 20 negative Spearman correlations

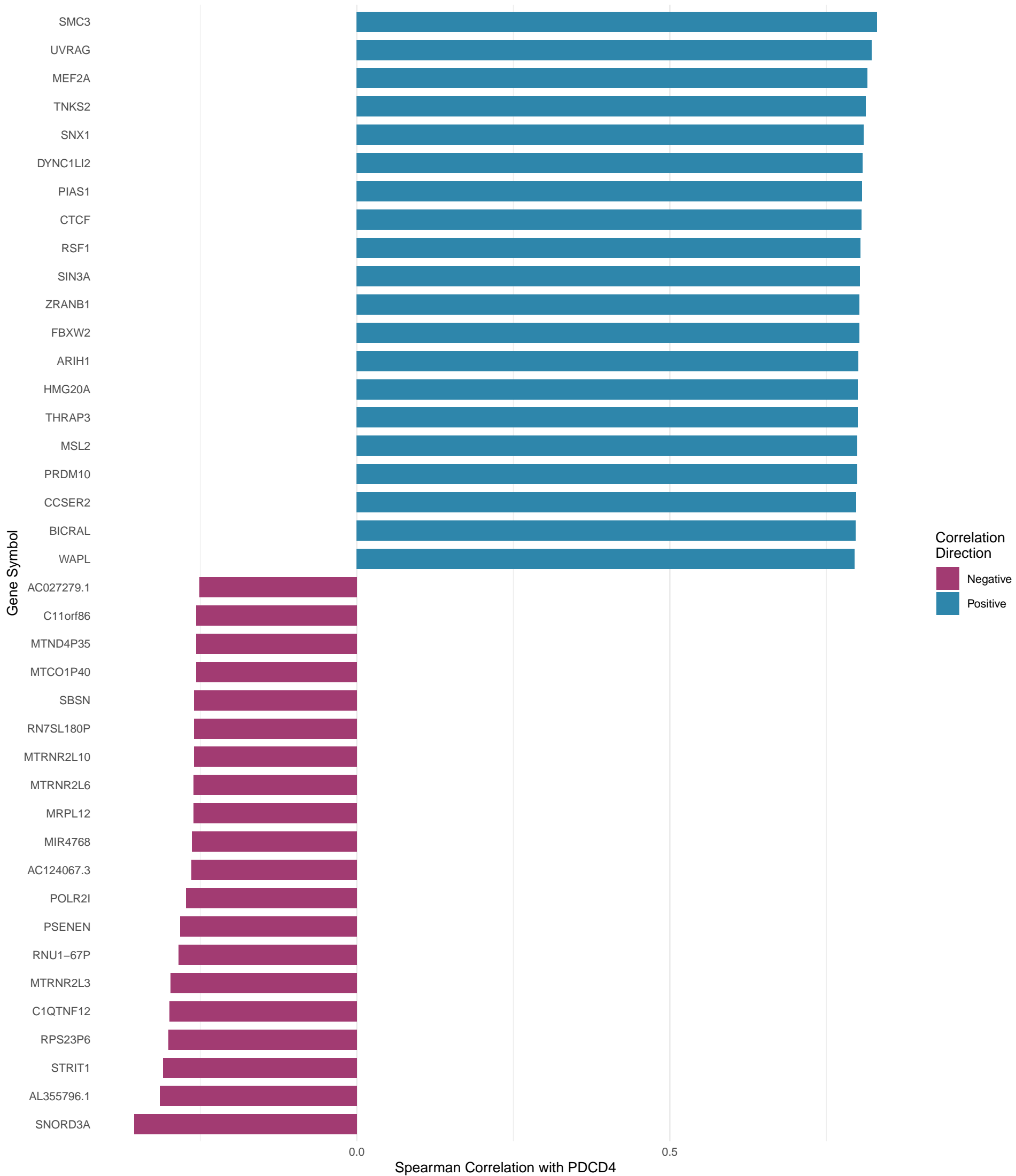

Supplement: Supplementary file 1 [file cimb-48-00022-s001.zip › Supp_Figure_S2.pdf]
